# Transposon mutagenesis of atypical enteroaggregative *Escherichia coli* reveals a hemagglutinin-associated protein that mediates cell adhesion and contributes to the *Galleria mellonella* virulence

**DOI:** 10.3389/fcimb.2023.1166158

**Published:** 2023-06-23

**Authors:** Mariane V. Monfardini, Renata T. Souza, Thais C. G. Rojas, Caroline G. Guerrieri, Cristina Orikaza, Isabel C. A. Scaletsky

**Affiliations:** ^1^ Departamento de Microbiologia, Imunologia e Parasitologia, Universidade Federal de São Paulo, Escola Paulista de Medicina, São Paulo, Brazil; ^2^ Departamento de Genética, Evolução e Bioagentes, Instituto de Biologia, Universidade Estadual de Campinas, Campinas, Brazil; ^3^ Departamento de Patologia, Centro de Ciências da Saúde, Universidade Federal do Espírito Santo, Vitória, Brazil

**Keywords:** enteroaggregative *E. coli* (EAEC), atypical EAEC, hemagglutinin, adhesion, *Galleria mellonella*

## Abstract

Twenty-two atypical enteroaggregative *Escherichia coli* isolates from a previous epidemiological study harboring EAEC virulence genes were examined for their adhesion properties. Nine strains showed a typical aggregative adherence (AA) pattern, while 13 strains showed variant AA, such as AA with lined up cells characteristic of the chain-like adhesion (CLA) and AA mainly to HeLa cells characteristic of the diffuse adherence (DA). The aggregative forming pilus (AFP) genes *afpA2* and *afpR* were detected only in strain Q015B, which exhibited an AA/DA pattern. Using Tn5-based transposon mutagenesis on Q015B strain, we identified a 5517-bp open reading frame (ORF) encoding a predicted 1838-amino-acid polypeptide that is genetically related to a putative filamentous hemagglutinin identified in *E. coli* strain 7-233-03_S3_C2. Therefore, the ORF was named *orfHA*. The regions flanking *orfHA* were sequenced and two ORFs were found; upstream, an ORF that encodes a 603-amino-acid polypeptide with 99% identity to hemolysin secretion/activation proteins of the ShlB/FhaC/HecB family, and downstream, another ORF, which encodes a 632-amino-acid polypeptide with 72% identity to the glycosyltransferase EtpC. An *orfHA* mutant (Q015BΔ*orfHA*) was constructed from strain Q015B. Q015BΔ*orfHA* strain did not adhere to HeLa cells, whereas Q015BΔ *orfHA* transformed with a pACYC184 plasmid carrying *orfHA* restored the AA/DA phenotype of strain Q015B. Furthermore, the Q015Δ*orfHA* mutant had a marked effect on the ability of strain Q015B to kill the larvae of *Galleria mellonella*. Our results suggest that the AA/DA pattern of strain Q015B is mediated by a hemagglutinin-associated protein which also contributes to its virulence in the *G. mellonella* model.

## Introduction

EAEC strains are defined by a characteristic “stacked-brick” aggregative adherence (AA) pattern to HEp-2 cells, which remains the gold standard for EAEC identification. The AA phenotype is mediated by aggregative adherence fimbriae (AAF) encoded by plasmids (pAA), and regulated by the transcriptional activator AggR, encoded on pAA plasmids (Nataro, 2003).

Strains containing the AggR regulon or its components are referred to as typical EAEC. In contrast, atypical EAEC strains lacking the AggR regulon and AAF genes are first considered avirulent (Nataro, 2003). However, epidemiological studies have shown that atypical (*aggR*-negative) EAEC strains can cause diarrhea (Jiang et al., 2002; Huang et al., 2007; Aslani et al., 2011; Boisen et al., 2012; Lozer et al., 2013). In addition, atypical EAEC caused two outbreaks of diarrhea (Cobeljic et al., 1996; Itoh et al., 1997), one of which affected more than 2,697 young children (Itoh et al., 1997).

Over the past decade, several advances have been made in defining the pathogenesis of EAEC, including the use of animal models, more recently the larvae of *Galleria mellonella* (Harrington et al., 2009; Roche et al., 2010; Jønsson et al., 2016; Khalil et al., 2016). Jønsson et al. (2016) and Khalil et al. (2016) analyzed typical EAEC strains in the *G*. *mellonella* model, compared them to non-pathogenic *E. coli*, and validated the model for EAEC pathogenicity studies. Previously, Guerrieri et al. (2019) showed that atypical EAEC strains were as virulent as typical EAEC strains in the *G. mellonella* model.

The mechanisms involved in the pathogenesis of atypical EAEC are largely unknown. An AAF-independent mechanism of adhesion in cultured cells and abiotic surfaces mediated by the IncI1 plasmid carrying type IV pili was described in the atypical EAEC strain C1096 (Dudley et al., 2006). Recently, a type IV pili called aggregate-forming pilus (AFP), encoded by a plasmid lacking *aggR* and containing the *aat* and *aap* genes, has been identified as responsible for the AA pattern of an EAEC/STEC (Shiga toxin-*E. coli*) hybrid strain. More recently, AFP was observed to mediate AA in a hybrid EAEC/UPEC (uropathogenic *E. coli*) strain (Schüroff et al., 2021; Schüroff et al., 2022). Dias et al. (2020) also observed the presence of *afp* genes only in atypical EAEC strains and suggested that these genes could be used to improve the diagnostic efficiency of atypical EAEC.

Previous studies have found high rates of asymptomatic EAEC infection among Brazilian children living in poor urban communities (Lima et al., 2000; Souza et al., 2009; Lima et al., 2013). In a previous epidemiological study in rural areas, we found a large number of atypical (*aggR*-negative) EAEC strains in children with and without diarrhea (Lozer et al., 2013). In this study, we included 22 atypical strains with EAEC virulence-related genes from our collection and assessed the presence of AFP genes, and examined their adhesion to cultured cells and abiotic surfaces. Furthermore, in this study we sought to identify the adhesins responsible for the AA phenotype of the *afp*-positive strain Q015B. Further studies revealed that the Q015B strain requires a putative filamentous hemagglutinin to adhere to HeLa cells, which may contribute to its virulence in the *G. mellonella* model.

## Materials and methods

### Bacterial strains and growth conditions

The 22 atypical (*aggR*-negative) EAEC strains used in this study were obtained from stool samples of children with diarrhea (five) and from healthy children (seventeen) in previous epidemiological studies conducted in rural areas of southeastern Brazil (Lozer et al., 2013). The strains were identified as EAEC by the AA pattern, and they all carried chromosomal and plasmid EAEC virulence genes (Mariane Monfardini, unpublished data) (**Table 1**). Strains were grown at 37°C in Luria–Bertani (LB) broth or on LB agar plates. Antibiotics were added to the medium at the following concentrations: kanamycin, 50 μg/mL; ampicillin, 100 μg/mL; and chloramphenicol, 25 μg/mL.

**Table 1:** Genotypic and phenotypic characteristics of 22 atypical EAEC.

Strain	Origin	EAEC virulence	Plasmid	Adherence	Biofilm	PCR results		
		genes	bands (n)	pattern	formation	*afpA*	*afpR*	*orfHA*
20A	Case	*pet, iucA*	>2	AA	+	–	–	–
39B	Control	*astA*	2	AA/DA	+	–	–	–
104A	Control	*pet, iucA*	>5	AA/CLA	+	–	–	–
133A	Control	*irp2*	>5	AA	–	–	–	–
Q015B	Case	*shf, astA, pet, aaiC, set1, iucA*	1	AA/DA	–	+	+	+
Q024A	Control	*pet, iucA*	>4	AA/DA	+	–	–	–
Q087A	Case	*irp2*	>5	AA/CLA	+	–	–	–
Q114A	Control	*pet, iucA*	1	AA/DA	–	–	–	-
Q158A	Control	*astA*	1	AA	–	–	–	–
Q165A	Control	*iucA*	>5	AA/CLA	+	–	–	–
Q181A	Control	*pet, iucA, chuA*	1	AA/CLA	–	–	–	–
Q185E	Control	*pet, irp2, iucA, chuA*	2	AA/DA	–	–	–	–
Q187B	Control	*shf, pet, irp2, iucA, chuA*	>4	AA/CLA	+	–	–	–
Q193A	Control	*shf, astA, irp2*	1	AA/DA	–	–	–	–
Q212D	Case	*pet, iucA*	1	AA/CLA	–	–	–	–
Q226A	Control	*astA, irp2*	2	AA	+	–	–	–
Q311A	Control	*pet, iucA, chuA*	2	AA/CLA	+	*-*	–	–
Q379B	Case	*astA*	>3	AA	+	–	–	–
Q385B	Control	*pet*	>5	AA	–	–	–	–
Q399A	Control	*pet, irp2, iucA*	>4	AA	+	–	–	–
Q440A	Control	*iucA*	2	AA	–	–	–	–
Q455A	Control	*shf, pet, irp2, iucA, chuA*	>5	AA	–	*-*	–	–

Case, children with diarrhea; Control, children without diarrhea; EAEC plasmid genes: shf criptic ORF; astA, heat-stable enterotoxin 1; pet, plasmid encoded toxin; EAEC chromosomal genes: aaiC, AaiC secreted protein; set1A, Shigella enterotoxin 1; iucA, aerobactin siderophore; chuA, E. coli haem utilization-gene. AA, aggregative adherence pattern; CLA, chain-like adherence pattern; DA, diffuse adherence pattern. (+), positive result; (-) negative result. (+), positive result; (-) negative result.

### DNA manipulation and genetic techniques

DNA manipulation was performed using standard laboratory protocols (Sambrook and Russell, 2001). Plasmid DNA was isolated using the QIAprep Spin Miniprep kit (Qiagen). Restriction endonucleases were used according to the manufacturer´s instructions (Invitrogen). Genomic DNA was isolated from non-adherent mutants using the PureLink Genomic DNA Mini kit (Invitrogen). PCR assay was performed using GoTaq Green Master Mix (Promega). The *afpA2* and *afpR* genes were amplified by PCR using primers as previously described (Dias et al., 2020). Eha ORF was examined by PCR using the primer sets: F1 (5´-CAACGGGAATGACAGCAACG-3´) and R1 (5´-CCGAGCTGTTTCTGCGGTAT-3´). DNA sequencing was performed at Centro de Estudos do Genoma Humano-USP in São Paulo.

### Transposon mutagenesis, screening, and rescue of interrupted genes

Transposon mutants of EAEC Q015B strain was obtained with kanamycin resistance (Km^R^)-encoding transposome EZ::TN <R6Kγ*ori*/KAN-2>Tnp transposome (Epicentre Biotechnologies) by electroporation according to the manufacturer´s protocol. Briefly, electrocompetent bacterial cells were transformed with 1 μL of Tnp transposome. Bacterial colonies grown on LB agar plates containing kanamycin were screened for adherence phenotype on HeLa cells as described below. Genomic DNA from non-adherent bacterial clones was digested with EcoRI and self-ligated by add T4 DNA ligase, and then used to transform *E. coli* DH5αΛ*pir*. The recovered DNA plasmids were purified and sequenced using transposon-specific primers R6KAN-2 RP-1 and KAN-2 FP-1 (Epicentre).

### Construction of an isogenic Q015BΔ*orfHA* mutant

A one-step PCR product inactivation method (Datsenko and Wanner, 2001) was used to construct the isogenic Q015BΔ *orfHA* mutant. PCR product amplified from pKD4 using primer pair F2 (5´-GTGAGCGGCCGGATGGCTGCCGCGCTTAGATTTATACGAGAATCATGCCGTGTAGGCGGAGCTGCTT-3´, underlined sequence, homologous to the kanamycin cassette of pKD4) and R2 (5´- GGGAGGTGTTCCTCCCCCGTCCCTCAGATTATGTCGCTAAAACAACAGGGCATATGAATATCCTCCTTAG-3´, underlined sequence, homologous to the kanamycin cassette of pKD4), flanked by the 5´and 3´ ends of *orfHA* with 50 bp of homology, was electroporated into strain Q015B carrying pKD46. Transformants were plated on LB agar containing kanamycin. Deletion of *orfHA* was confirmed by PCR using primers F1 and R1 and F3 (5´-GAGCAGAACCGCAACAACAC-3´) and R3 (5´- ATGCACAAGTGCTTCAAAGGT-3´).

### Construction of *orfHA* complementation plasmid

The *orfHA* was generated by PCR from Q015B genomic DNA using primer pairs F4 (5´-TATTGTCGACTCAGATTTATACGAGAATCATGCC-3´, underlined sequence, SalI/HindIII sites) and R4 (5´-TATTAAGCTTTCCCCCGTCCCTCAGATTAT-3´, underlined sequence, SalI/HindIII sites. The resultant 5.5 kb gel-purified PCR product was then cloned into the SalI and HindIII sites of pACYC184 to generate plasmid pMM2 and transformed into the Q015BΔ*orfHA* mutant. Transformants were plated on LB agar containing chloramphenicol.

### HeLa cell adherence assay

HeLa adherence assay was modified as previously described (Scaletsky et al., 1984). Briefly, monolayers of 10^5^ HeLa cells were cultured in Dulbecco´s Modified Eagle´s Medium (DMEM) containing 10% fetal bovine serum using 24-well tissue culture plates containing 9-mm diameter glass coverslips. Bacterial strains were grown statically in 2 mL LB at 37°C for 16-18 h. Cell monolayers were infected by adding 20 μL of bacterial cultures to 1 mL DMEM containing 1% D-mannose and were incubated at 37°C for 3 h. The presence of mannose in the assay inhibits the adhesion of type 1 pili. After incubation, cells were washed with sterile PBS, fixed with methanol, stained with Giemsa dye, and examined under light microscopy. To quantify bacterial adhesion to HeLa cells, the washed monolayers were lysed in 1% Triton X-100 for 30 min at room temperature. Serial dilutions of lysate from each well was plated onto LB agar plates, with selective antibiotics where appropriate, to obtain viable counts.

### Biofilm assay

Biofilm assay was performed as previously described by Sheikh et al. (2001). Briefly, bacterial strains were grown in 2 mL LB at 37°C for 16-18 h. Aliquots of 5 μL of bacterial cultures were grown in DMEM supplemented with 0.45% glucose in 96-well flat-bottom polystyrene plates. The culture plates were incubated overnight at 37°C. The medium was then aspirated, and the substrate washed three times with PBS. Biofilms were visualized by staining with 0.5% crystal violet for 10 min, washing twice with PBS, and air drying. Biofilm formation was quantified by an ELISA plate reader at 570 nm (DO_570_) by adding 1 ml of 95% ethanol to the wells to solubilize crystal violet. EAEC 042 was used as a positive control and *E. coli* HB101 was used as a negative control. Data analyses were performed according to the criteria of Stepanovic et al. (2007).

### Hemagglutination assay

Fresh erythrocytes were obtained from human type A, sheep, rabbit, guinea pig and bovine. Strains were grown overnight in LB without shaking. An aliquot of 100 μL of each bacterial culture was added to 10 mL of High-Glucose DMEM and incubated at 37°C for 4 h; bacteria were pelleted by centrifugation, and then washed twice in sterile PBS. Pellet was resuspended in PBS at a concentration of 10^8^ CFU/mL (estimated by McFarland scale). Twofold serial dilutions of bacterial suspensions in PBS were prepared. An aliquot of 100 μL of each dilution was mixed with equal volume of a 3% (vol/vol) erythrocytes suspension containing 1% D-mannose in 96-well round bottom polystyrene plates and let stand at 4°C for 20 min. HA was evaluated under an inverted microscope at 20X magnification.

### *Galleria mellonella* infection assay

The *Galleria mellonella* assay was carried out according to Ramarao et al. (2012). Bacterial cultures at mid-exponential phase were injected into *G. mellonella* larvae at a concentration of 10^5^ CFU/larva. Briefly, 10 μL aliquots of bacterial suspension were injected into 20 final instar larvae. Infected larvae were placed in dish plates and incubated at 37°C, monitoring mortality every 24 h until 96 h post-infection. Larvae that did not respond to touch were considered dead. Negative controls included larvae inoculated with sterile PBS and non-pathogenic *E. coli* strain HB101. Infection assays were performed in triplicate on different days. Strains were classified as highly virulent if less than 40% of larvae survived, and as moderately or lowly virulent if 40 to 60% or greater than 60% of larvae survived at the end of the assay.

### Data and statistical analyses

All *in vitro* assays were performed in triplicate with results from at least three separate assays. The results of adhesion and biofilm formation were compared using a Student´s *t*-test. To analyze virulence in *G. mellonella* model, survival curves were made using the Kaplan-Meier method. The Log-Rank test was performed to observe if there was a significant difference in survival curves and Student´s t-test was performed to analyze the survival percentage after 96 h. All analyses were performed using Graphpad Prism 6 and the degree of statistical significance considered was 95% (p < 0.05).

## Results

**Figure 1:**
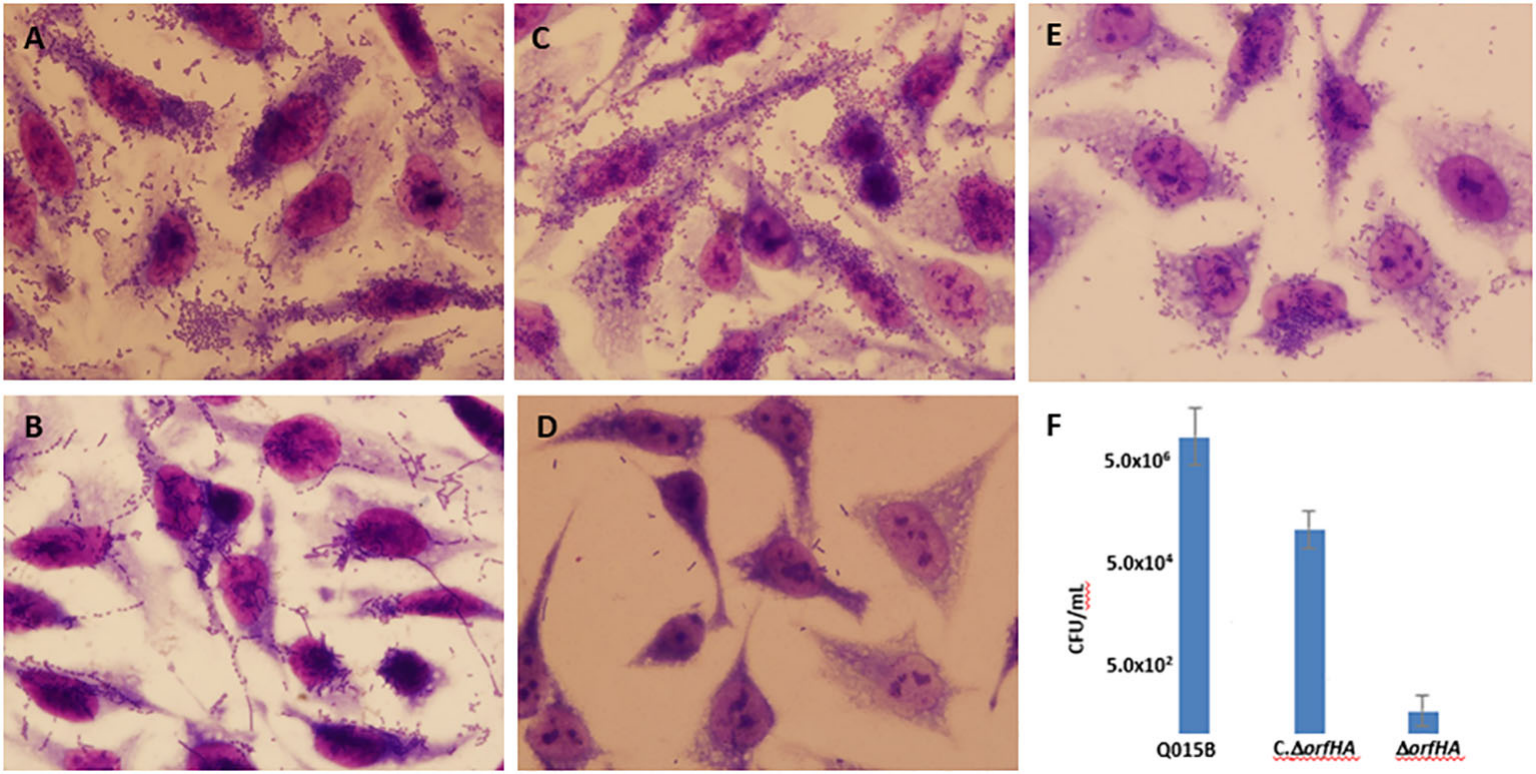
Representative images of aggregative adhesion (AA) variants of atypical EAEC strains on HeLa cells after 3 h. **(A)** typical AA pattern; **(B)** AA pattern with chain-like aggregates (CLA); **(C)** AA with diffuse adhesion (DA) shown in strain Q015B; **(D)** and **(E)** Adhesion of Q015BΔorfHA mutant and complemented Q015BC.ΔorfHA strains. Magnification, x1,000. **(F)** Quantitative of adherence of Q015B, mutant and complemented strains. Serial dilutions of lysate from each well onto LB agar plates, with selective antibiotics where appropriate, to obtain viable counts.

The results of the HeLa adherence and biofilm assays are shown in **Table 1**. Among the 22 atypical EAEC strains, 9 strains showed typical AA with bacterial aggregates attached to HeLa cells and coverslips (**Figure 1A**), and the remaining strains showed variations of the AA pattern. Seven strains showed AA pattern with chain-like aggregates (CLA) on HeLa cells and coverslips (**Figure 1B**), and six strains showed AA, with bacteria predominantly adhering to HeLa cells, characteristic of the diffuse adhesion (DA) pattern (**Figure 1C**). AA patterns were found in EAEC strains isolated from both cases and controls. Strains were tested for their ability to form biofilms in microtiter plates, and 11 strains were able to form biofilms. The 11 biofilm-producing strains were classified as strong (OD_570_ ≥ 1.0) and weak (OD_570_ > 0.5 < 1) biofilm producers compared to the EAEC 042 strain (OD_570_ ≥ 1.0) (**Figure S1**).

We used PCR to determine the presence of genes associated with the newly described AFP adhesin. AFP-encoding genes (*afpA2* and *afpR*) were only detected in strain Q015B. This strain showed an AA/DA pattern (**Figure 1C**) and carried plasmid genes (*shf*, *astA*, and *pet*) and chromosomal genes (*aaiC* and *set1A*) (**Figure S2**).

To identify AA adhesins from the Q015B strain, a Tn5 transposon insertion library was constructed. A total of 1,200 independent-inserted mutant strains were tested for their ability to adhere to HeLa cells and 15 mutants were found to be defective in adherence; all mutants showed growth rates comparable to the parental strain (**Figure S3**). The transposon insertion site of each mutant was cloned and the regions flanking the insertion were sequenced.

**Figure 2:**
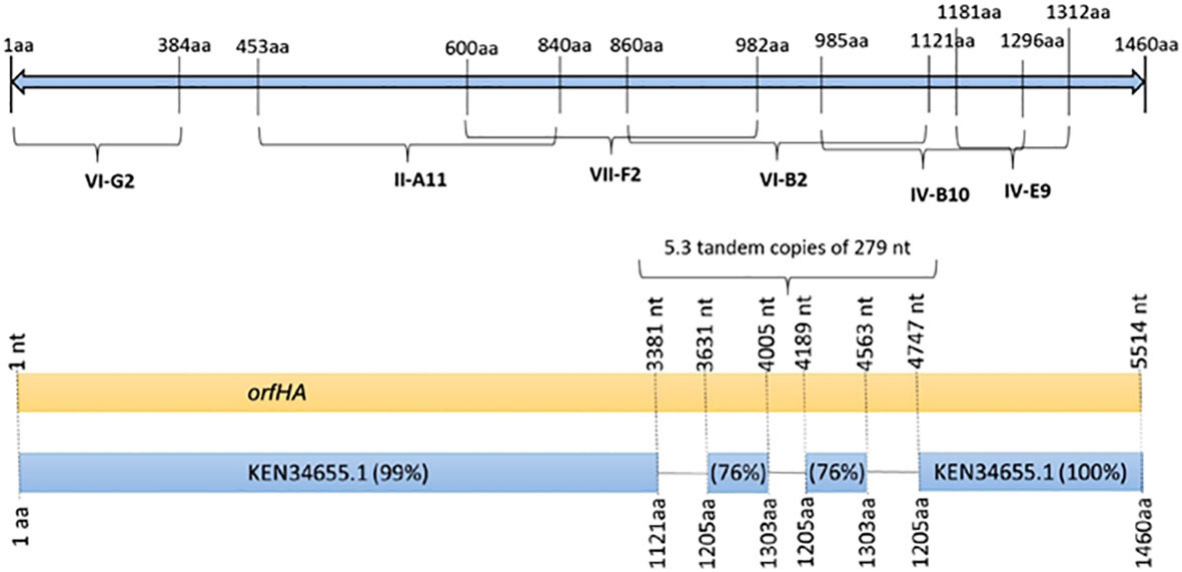
Schematic representation of the 1460 amino acid filamentous hemagglutinin from *E. coli* strain 7-233-03_S3_C2 (GenBank accession KEN34655.1) showing the presence of transposon insertion sites in mutants VI-G2, II-A11, VII-F2, VI-B2, IV-B10, and IV-E9. Schematic diagram of *orfHA* determined by nucleotide sequence showing percent identity to filamentous hemagglutinin.

In three mutants, the transposon insertion site was too short for analysis. Six transposon insertions were found in genes related to transcriptional regulators, amino acid biosynthesis, transposases, DNases, or transport systems. Six other mutants (II-A11, IV-B10, IV-E9, VI-B2, VI-G2, and VII-F2) were found to have transposon insertions at different sites of the same 4,380-bp open reading frame (ORF) encoding a filamentous hemagglutinin of *E. coli* strain 7-233-03_S3_C2 (GenBank accession no. KEN34655.1) (**Figure 2**). Therefore, we named *orfHA*. Using primers obtained from sequences adjacent to the transposon insertion and primer walk, we determined the entire sequence of the disrupted region in the Q015B strain, Sequence analysis of *orfHA* revealed a 5517-bp ORF encoding a predicted 1838-amino-acid polypeptide wich displayed 99 and 100% homology to the first 1121 amino acids (nt 1 to 3381) and last 255 amino acids (nt 4747 to 5517) with the filamentous hemagglutinin identified in *E. coli* strain 7-233-03_S3_C2; between nucleotides 3381 and 4747 were found two repeats which displayed 76% homology with amino acids 1205 to 1303. Analysis of these regions with the Tandem Repeats Finder program revealed 5.3 tandem copies of 279 nucleotides (**Figure 2**).

Further sequencing of the flanking regions of *orfHA* revealed two candidate ORFs in the same orientation. The upstream ORF encodes a predicted 603-amino-acid polypeptide with 99% identity to a hemolysin secretion/activator protein of the ShlB/FhaC/HecB family [*Escherichia coli*] (GenBank accession no. WP_077763896.1) and 51% identity to afimbrial adhesin Enf from EAEC O111:H12 (GenBank accession no. AAK58509.1), and 49% with the two-partner secretion system transporter EtpB [*Escherichia coli*] (GenBank accession no. WP_000260524.1). The downstream ORF encodes a predicted 632-amino-acid polypeptide with 72% identity to the glycosyltransferase EtpC (GenBank accession no. KEM44334.1). Two additional non-adherent mutants (II-D1 and VIII-A11) were found at different sites in the upstream ORF.

To determine whether *orfHA* plays a role in the adhesion of Q015B to HeLa cells, we constructed an isogenic *orfHA* deletion mutant described in Materials and Methods. The resulting mutant Q015BΔ*orfHA* strain was verified by PCR (**Figure S4**). Mutant Q015BΔ*orfHA* did not adhere to HeLa cells (**Figure 1D**), whereas transformation of Q015BΔ*orfHA* with recombinant pACYC184 plasmid (pMM2) carrying only *orfHA* restored the AA/DA phenotype of strain Q015B (**Figure 1E**). As shown in **Figure 1**, the number of bacteria adhered to HeLa cells was significantly reduced in the orfHA mutant compared with the Q015B strain. The complemented strain partly restored the bacterial adhesion. These results indicate that *orfHA* is required for Q015B to adhere to HeLa cells. To determine the prevalence of *orfHA* in the other 21 strains, primers were deduced from the nucleotide sequences and PCRs were performed. No strain was *orfHA*-positive.

Q015B was found to carry a large plasmid called pMM1, so we tested whether this plasmid encodes an adhesion factor. Plasmid DNA was extracted from strain Q015B containing Tn5 kanamycin, purified through a CsCl2 gradient and grown in transformed *E. coli* DH5α. *E. coli* DH5α transformants harboring pMM1 were screened for their ability to adhere to HeLa cells, but none adhered (**Figure S5**). Eletroporation was used to cure the pMM1 plasmid from Q015B strain. After electroporation, bacterial colonies were analyzed for the presence of the pMM1 plasmid. Strain Q015B-1 resulted in the loss of the pMM1 plasmid but had no effect on adhesion of strain Q015B (**Figure S5**). These data suggest that the genes responsible for the AA/DA phenotype of strain Q015B are found on the bacterial chromosome. These findings indicate that the *orfHA* involved in the adhesion of Q015B to HeLa cells is not plasmid-encoded.

Next, we examined the hemagglutination (HA) activity of erythrocytes from different animal species and verified that the Q015B strain displayed HA on bovine, sheep, rabbit, guinea pig, and human erythrocytes, whereas the Q015BΔ*orfHA* mutant strain showed no HA activity (**Figure S6**). The HA activity of the Q015BΔ*orfHA* complemented strain was similar to that of the Q015B strain.

**Figure 3:**
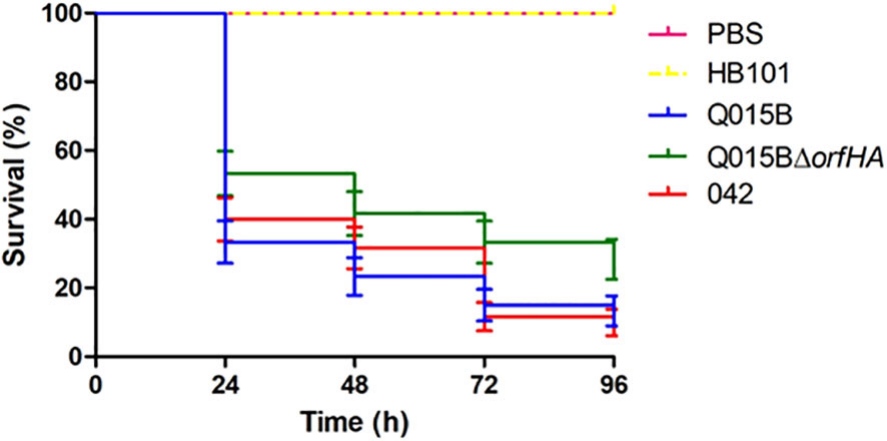
Survival curve graph of *Galleria mellonella* infected with 10^5^ cells of Q015B and Q015BΔ*orfHA* strains. The line represents the average of three independent experiments performed with monitoring mortality. The difference between Q015B and Q015Δ*orfHA* mortality was significant (*P* = 0.02).

The Q015B strain has previously been shown to be virulent in the *Galleria melonella* model (Guerrieri et al., 2019). To investigate whether *orfHA* is involved in this phenotype, we tested the strain Q015BΔ*orfHA* in the *G. mellonella* model (**Figure 3**). Compared with the Q015B strain, the mortality rate of larvae inoculated with Q015BΔ*orfHA* was significantly reduced (P<0.02). Only 13.3% of larvae inoculated with the Q015B strain survived the first 24 hours, compared with 28.3% of larvae inoculated with the Q015BΔ*orfHA* mutant strain (**Figure 3**).

## Discussion

EAEC can be distinguished from other diarrheagenic *E. coli* by its aggregative adherence (AA) pattern to cultured epithelial cells and glass coverslips (Nataro, 2003). In typical EAEC (*aggR*-positive), AAF is required for the AA phenotype, but other factors, including afimbrial adhesins, are also associated with this phenotype (Monteiro-Neto et al., 2003; Dudley et al., 2006). To characterize AA factors, 22 atypical EAEC (*aggR*-negative) strains isolated in previous epidemiological studies (Lozer et al., 2013) and carrying EAEC virulence genes were selected for further characterization. Of the 22 strains examined, 9 strains showed the typical AA pattern on HeLa cells, 7 strains displayed both AA and CLA (AA/CLA), and 6 strains displayed AA/DA without forming aggregates on coverslips. Variations in AA patterns have been described, including bacteria that primarily display AA on coverslips or epithelial cells (Knutton et al., 1992; Gioppo et al., 2000). Previously, a Brazilian study reported that typical EAEC strains displayed an AA/CLA or DA pattern on HeLa cells (Dias et al., 2020). Another study from Brazil identified an atypical EAEC strain exhibiting a localized adherence (LA) pattern (Freire et al., 2022).

More recently, Lang et al. (2018) described a new type of pili called aggregate-forming pili (AFP), in a mixed EAEC/STEC serotype 023:H18 isolate lacking AAF. Deletion of the entire *afp* operon, results in reduced pili production and reduces the ability of EAEC/STEC isolates to adhere to epithelial cells (Lang et al., 2018). In this study, we checked for the presence of the *afp* genes and found that only the Q015B strain was positive for *afpA2* and *afpR* genes.

To identify the gene responsible for the AA phenotype, we performed transposon mutagenesis in strain Q015B and screened for adhesion-deficient mutants. In this study, we identified a filamentous hemagglutinin-associated protein in strain Q015B, which we named *orfHA*. Subsequent sequence and deletion analyzes and complementation studies confirmed that strain Q015B required *orfHA* for cell adhesion.

The *orfHA* flanking region in the Q015B strain has been sequenced and revealed two ORFs in the same transcriptional orientation. Interestingly, two transposon insertion sites are located in the upstream region. These findings suggest that these two ORFs may be involved in the secretion of *orfHA*, representing a potential two-partner secretion locus. A number of potential two-partner secretion pathway (TPS) genes have been identified in genome sequences of various pathogens (Jacob-Dubuisson et al., 2004). Fleckenstein et al. (2006) identified a two-partner secretion locus (etpBAC) on the pCS1 virulence plasmid of enterotoxigenic *E. coli* strain H10407.

The ability of EAEC strains to kill *G. mellonella* larvae has not been found to be associated with EAEC virulence genes (Guerrieri et al., 2019). Interestingly, the Q015BΔ*orfHA* mutant strain showed significantly reduced mortality in inoculated larvae compared to the Q015B strain. These results suggest that *orfHA* may contribute to larval mortality. Reduced larval mortality was observed with the EAEC 042 *aggR* mutant strain compared to the wild-type strain (Guerrieri et al., 2019). According to these authors, the virulence of EAEC appears to be related to AggR, but not completely, and atypical EAEC strains may be as virulent as typical strains in the *G. mellonella* model.

Two previous studies reported plasmid-encoded outer membrane proteins associated with adhesion and hemagglutination properties of EAEC strains. Yamamoto et al. (1996) reported that *E. coli* O73:H33 adhesion was mediated by a 110 kbp plasmid and was associated with mannose-resistant hemagglutinin detected with bovine, sheep, or human erythrocytes. Monteiro-Neto et al. (2003) described a plasmid-encoded 58 kDa outer membrane protein associated with hemagglutination and adhesion properties of the EAEC-O111:H12 strain. Plasmid analysis revealed that strain Q015B contained a large plasmid, designated pMM1, and the possibility of an adhesion factor encoded by the plasmid was investigated. In this study, we found that *orfHA* in the strain Q015B was not plasmid-encoded but detected with bovine, sheep or human erythrocytes.

Taken together, our results suggest that the AA/DA pattern of strain Q015B may be mediated by a hemagglutinin-associated protein, which may contribute to its virulence in the *G. mellonella* model. Future work aims to fully characterize this hemagglutinin.

## Data availability statement

The original contributions presented in the study are included in the article/**Supplementary Material**. Further inquiries can be directed to the corresponding author.

## Author contributions

IS and MM named the study. MM and CG performed the experiments. MM, CG, RS and TR analyzed the data. IS wrote the manuscript. CO helped with the HeLa cells and photomicrographs. All authors contributed to the article and approved the submitted version.

## References

[B1] Aslani M. M. Alikhani M. Y. Zavari A. Yousefi R. Zamani A. R. (2011). Characterization of enteroaggregative Escherichia coli (EAEC) clinical isolates and their antibiotic resistance pattern. Int. J. Infect. Dis. 15, e136–e139. doi: 10.1016/j.ijid.2010.10.002 21130676

[B2] Boisen N. Scheutz F. Rasko D. A. Redman J. C. Persson S. Simon J.. (2012). Genomic characterization of enteroaggregative Escherichia coli from children in Mali. J. Infect. Dis. 205, 431–444. doi: 10.1093/infdis/jir757 22184729 PMC3256949

[B3] Cobeljic M. Miljkovic-Selimovic B. Paunovic-Todosijevic D. Velickovic Z. Lepsanovic Z. Zec N.. (1996). Enteroaggregative *Escherichia coli* associated with an outbreak of diarrhea in a neonatal nursery ward. *Epidemiol* . Infect 117, 11–16. doi: 10.1017/s0950268800001072 PMC2271665 8760945

[B4] Datsenko K. A. Wanner B. L. (2001). One-step inactivation of chromossomal genes in *Escherichia coli* K-12 using PCR products. proc. natl. Acad. Sci. U.S.A. 97, 6640–6645. doi: 10.1073/pnas.120163297 PMC18686 10829079

[B5] Dias R. C. B. Tanabe R. H. S. Vieira M. A. Cergole-Novella M. C. Dos Santos L. F. Gomes T. A. T.. (2020). Analysis of the virulence profile and phenotypic features of typical and atypical enteroaggregative *Escherichia coli* (EAEC) isolated from diarrheal patients in Brazil. Front. Cell. Infect. Microbiol. 10. doi: 10.3389/fcimb.2020.00144 PMC7188757 32391284

[B6] Dudley E. G. Abe C. Ghigo J. Latour-Lambert P. Hormazabal J. C. Nataro J. P. (2006). An IncI1 plasmid contributes to the adherence of the atypical enteroaggregative *Escherichia coli* strain C1096 to cultured cells and abiotic surfaces. Infect. Immun. 74, 2102–2114. doi: 10.1128/IAI.74.4.2102-2114 16552039 PMC1418932

[B7] Fleckenstein J. M. Roy K. Fischer J. F. Burkitt M. (2006). Identification of a two-partner secretion locus of enterotoxigenic *Escherichia coli* . Infect. Immun. 74, 2245–2258. doi: 10.1128/IAI.74.4.2245-2258.2006 16552055 PMC1418895

[B8] Freire C. A. Rodrigues B. O. Elias W. P. Abe C. M. (2022). Adhesin related genes as potential markers for the enteroaggregative *Escherichia coli* category. Front. Cell. Infect. Microbiol. 12. doi: 10.3389/fcimb.2022.997208 PMC9679366 36425788

[B9] Gioppo N. M. R. Elias W. P. Vidotto M. C. Linhares R. E. Saridakis H. O. Gomes T. A.. (2000). Prevalence of HEp-2 cell-adherent *Escherichia coli* and characterization of enteroaggregative *E. coli* and chain-like adherent *E. coli* isolated from children with and without diarrhea, in londrina, Brazil. FEMS Microbiol. Lett. 190, 293–298. doi: 10.1111/j.1574-6968 11034294

[B10] Guerrieri C. G. Pereira M. F. Galdino A. C. M. Dos Santos A. L. S. Elias W. P. Schuenck R. P.. (2019). Typical and atypical enteroaggregative *Escherichia coli* are both virulent in the *Galleria mellonella* model. Front. Microbiol. 10. doi: 10.3389/fmicb.2019.01791 PMC6700222 31456762

[B11] Harrington S. M. Sheikh J. Henderson I. R. Ruiz-Perez F. Cohen P. S. Nataro J. P.. (2009). The Pic protease of enteroaggregative Escherichia coli promotes intestinal colonization and growth in the presence of mucin. Infect. Immun. 77, 12–2465–2473. doi: 10.1128/IAI.01494-08 PMC2687332 19349428

[B12] Huang D. B. Mohamed J. A. Nataro J. P. DuPont H. L. Jiang Z. D. Okhuysen P. C. (2007). Virulence characteristics and the molecular epidemiology of enteroaggregative *Escherichia coli* isolates from travelers to developing countries. J. Med. Microbiol. 56, 1386–1392. doi: 10.1099/jmm.0.47161-0 17893178

[B13] Itoh Y. Nagano I. Kunishima M. Ezaki T. (1997). Laboratory investigation of enteroaggregative *Escherichia coli* O untypeable:H10 associated with a massive outbreak of gastrointestinal illness. J. Clin. Microbiol. 35, 2546–2550. doi: 10.1128/jcm.35.10.2546-2550 9316905 PMC230008

[B14] Jacob-Dubuisson F. Fernandez R. Coutte L. (2004). Protein secretion through autotransporter and two-partner pathways. Biochim. Biophys. Acta 1694, 235–257. doi: 10.1016/j.bbamcr.2004.03.008 15546669

[B15] Jiang Z. D. Greenberg D. Nataro J. P. Steffen R. DuPont H. L. (2002). Rate of occurrence and pathogenic effect of enteroaggregative *Escherichia coli* virulence factors in intestinal travelers. J. Clin. Microbiol. 40, 4185–4190. doi: 10.1128/JCM.41.5.2138-2140 12409395 PMC139678

[B16] Jønsson R. Struve C. Jenssen H. Krogfelt K. A. (2016). The wax moth *Galleria mellonella* as a novel model system to study enteroaggregative *Escherichia coli* pathogenesis. Virulence 8, 1894–1899. doi: 10.1080/21505594.2016.1256537 PMC5810504 27824518

[B17] Khalil U. Younus M. Asghar N. Siddiqui F. Gomez-Duarte O. G. Wren B. W.. (2016). Phenotypic and genotypic characterization of enteroaggregative *Escherichia* coli isolates from pediatric population in Pakistan. APMIS 7, 872–880. doi: 10.1111/apm.12577 27485156

[B18] Knutton S. Sham R. K. Bhan M. K. Smith H. R. McConnell M. M. Cheasty T.. (1992). Ability of enteroaggregative *Escherichia coli* strains to adhere *in vitro* to human intestinal mucosa. Infect. Immun. 60, 2083–2091. doi: 10.1128/iai.60.5.2083-2091.1992 1348724 PMC257118

[B19] Lang C. Fruth A. Holland G. Laue M. Mũhlen S. Dersch P.. (2018). Novel type of pilus associated with a shiga-toxigenic *E. coli* hybrid pathovar conveys aggregative adherence and bacterial virulence. Emerg. Microbes Infect. 7, 203. doi: 10.1038/s41426-018-0209-8 30514915 PMC6279748

[B20] Lima I. F. Boisen N. Quetz Jda S. Havt A. de Carvalho E. B. Soares A. M.. (2013). Prevalence of enteroaggregative *Escherichia coli* and its virulence related genes in a case-control study among children from north-eastern Brazil. J. Med. Microbiol. 62, 683–693. doi: 10.1099/jmm.0.054262-0 23429698 PMC3709657

[B21] Lima A. A. M. Moore S. R. Barbosa M. S. Jr. Soares A. M. Schleupner M. A. Newman R. D.. (2000). Persistent diarrhea signals a critical period of increased diarrhea burdens and nutritional shortfalls: a prospective cohort study among children in northeatern Brazil. J. Infect. Dis. 181, 1643–1651. doi: 10.1086/315423 10823764

[B22] Lozer D. M. Souza T. B. Monfardini M. V. Vicentini F. S. Kitagawa S. Scaletsky I. C. A.. (2013). Genotypic and phenotypic analysis of diarrheagenic *Escherichia coli* strains isolated from Brazilian children living in low socioeconomic level communities. BMC Infect. Dis. 13, 418. doi: 10.1186/1471-2334-13-418 24010735 PMC3846636

[B23] Monteiro-Neto V. Bando S. Y. Moreira-Filho C. A. Girón J. A. (2003). Characterization of an outer membrane protein associated with haemagglutination and adhesive properties of enteroaggregative *Escherichia coli* O111:H12. Cell Microbiol. 5, 533–547. doi: 10.1046/j.1462-5822.2003.00299.x 12864813

[B24] Nataro J. P. (2003). “Enteroaggregative escherichia coli,” in Emerging infections 6. Eds. Scheld W. M. Hughes J. M. Murray B. E. (Washington, D.C: ASM Press).

[B25] Ramarao N. Nielsen-Leroux C. Lereclus D. (2012). The insect *Galleria mellonella* as a powerful infection model to investigate bacterial pathogenesis. J. Vis. Exp. 70, 1–7. doi: 10.3791/4392 PMC3567165 23271509

[B26] Roche J. K. Cabel A. Servilleja J. Nataro J. Guerrant R. L. (2010). Enteroaggregative *Escherichia coli* (EAEC) impairs growth while malnutrition worsens EAEC infection: a novel murine model of the infectious malnutrition cycle. J. Infect. Dis. 22908, 506–514. doi: 10.1086/654894 PMC2919845 20594107

[B27] Sambrook J. Russell D. W. (2001). Molecular cloning: a laboratory manual. 3rd ed (Cold Spring Harbor, N.Y: Cold Spring Harbor Laboratory Press).

[B28] Scaletsky I. C. A. Silva M. L. Trabulsi L. R. (1984). Distinctive patterns of adherence of enteropathogenic *Escherichia coli* to HeLa cells. Infect. Immun. 45, 534–536. doi: 10.1128/IAI.45.2.534-536.1984 6146569 PMC263286

[B29] Schüroff P. A. Abe C. M. Silva J. W. Coelho C. P. Andrade F. B. Hernandes R. T.. (2022). Role of aggregate-forming pilus (AFP) in adherence and colonization of both intestinal and urinary tracts. Virulence 13, 1423–1433. doi: 10.1080/21505594.2022.2112818 35982607 PMC9397481

[B30] Schüroff P. A. Salvador F. A. Abe C. M. Wami H. T. Carvalho E. Hernandes R. T.. (2021). The aggregate-forming pili (AFP) mediates the aggregative adherence of a hybrid-pathogenic escherichia coli (UPEC/EAEC) isolated from a urinary tract infection. Virulence 12, 3073–3093. doi: 10.1080/21505594.2021.2007645 34923895 PMC8923075

[B31] Sheikh J. Hicks S. Dall´Agnol M. Phillips A. D. Nataro J. P. (2001). Roles for Fis and YafK in biofilm formation by enteroaggregative *Escherichia coli* . Mol. Microbiol. 41, 983–997. doi: 10.1046/j.1365-2958.2001.02512.x 11555281

[B32] Souza T. B. Morais M. B. Tahan S. Melli C. F. L. Rodrigues M. S. C. Scaletsky I. C. A. (2009). High prevalence of antimicrobial drug-resistant diarrheagenic *Escherichia coli* in asymptomatic children living in an urban slum. J. Infect. 59, 247–251. doi: 10.1016/j.jinf.2009.08.007 19706305

[B33] Stepanovic S. Vukovic D. Hola V. Di Bonaventura G. Djukic S. Crikovic I.. (2007). Quantification of biofilm in microtiter plates: overview of testing conditions and practical recommendations for assessment of biofilm production by staphylococci. APMIS 115, 891–899. doi: 10.1111/j.1600-0463.2007.apm.630.x 17696944

[B34] Threlfall E. J. Rowe B. Ferguson J. L. Ward L. R. (1986). Characterization of plasmids conferring resistance to gentamicin and apramycin in strains of *Salmonella typhimurium* phage type 204c isolated in Britain. J. Hyg. (Lond.) 97, 419–426. doi: 10.1017/s0022172400063609 3540111 PMC2082898

[B35] Yamamoto T. Wakisaka N. Nakae T. Kamano T. Serichantalergs O. Echeverria P. (1996). Characterization of a novel hemagglutinin of diarrhea-associated *Escherichia coli* that has characteristics of diffusely adhering *E. coli* and enteroaggregative *E. coli* . Infect. Immun. 64, 3694–3702. doi: 10.1128/iai.64.9.3703-3712.1996 8751919 PMC174283

