# Supplementary material for: Transposon mutagenesis of atypical enteroaggregative Escherichia coli reveals a hemagglutinin-associated protein that mediates cell adhesion and contributes to the Galleria mellonella virulence
Source: Front Cell Infect Microbiol. 2023 Jun 23;13:1166158. doi: 10.3389/fcimb.2023.1166158 (PMC10327481; doi:10.3389/fcimb.2023.1166158)
Supplement: Supplementary file 1 [file DataSheet_1.pdf]

Supplementary Material

Transposon mutagenesis of atypical enteroaggregative *Escherichia coli* reveals a hemagglutinin-associated protein that mediates cell adhesion and contributes to the *Galleria mellonella* virulence

Mariane V. Monfardini, Renata T. Souza, Thais C. G. Rojas, Caroline G. Guerrieri, Cristina M. O. Toqueiro and Isabel C. A. Scaletsky*

*** Correspondence:** Address for correspondence: Isabel C A Scaletsky, PhD, Departamento de Microbiologia, Imunologia e Parasitologia, Universidade Federal de São Paulo, Escola Paulista de Medicina, Rua Botucatu, 862, 3 andar, 04023-062, São Paulo, Brazil. Tel.: +55 11 55764848; E-mail: scaletsky@unifesp.br

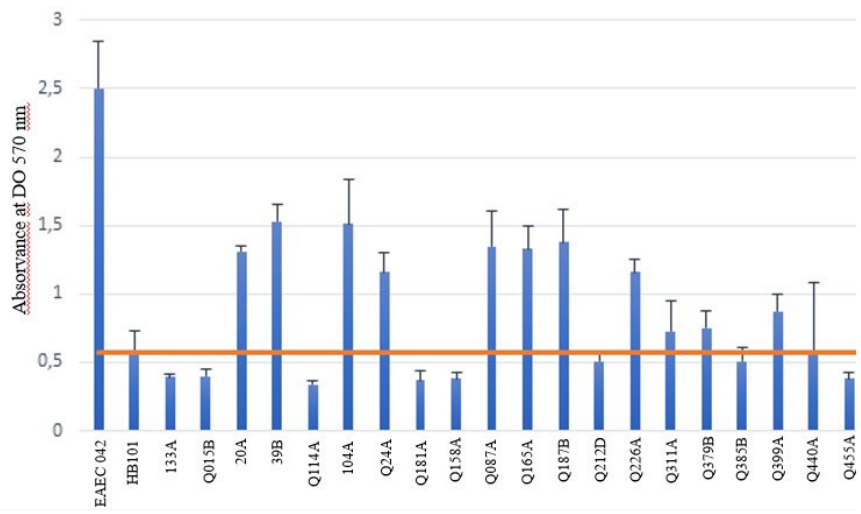

FIGURE S1. Quantification of biofilm formation by 20 atypical EAEC strains. Bacteria were cultivated in DMEM-0.45% glucose for 24 h at 37°C in 24- well dishes. Biofilms were fixed and stained with crystal violet, and then the strains were solubilized and quantitated spectrophotometrically at 570 nm. The bars represent the means of the results from triplicate wells; error bars indicate one standard deviation. The line represents the threshold for classification of biofilm producers.

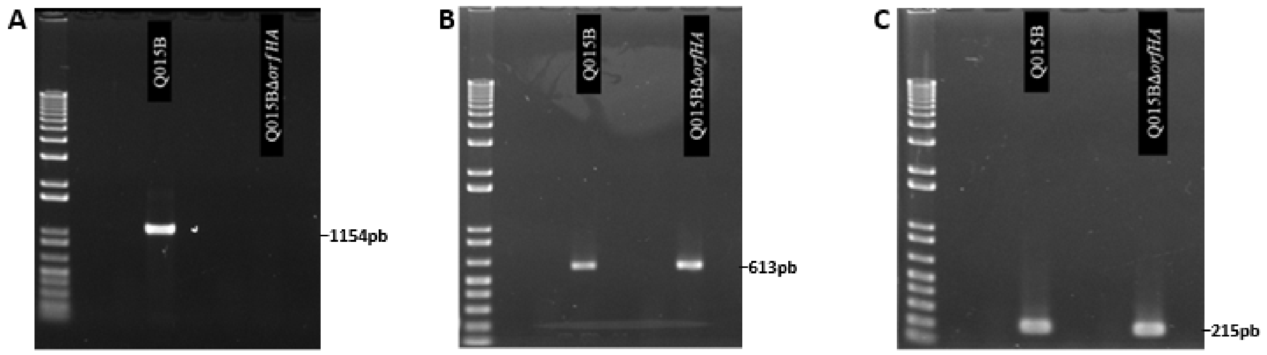

FIGURE S2. Detection of virulence genes in EAEC Q015B and Δ*orfHA* mutant strains by RT-PCR. RNA was extracted from EAEC Q015B and Δ*orfHA* mutant subjected to reverse transcriptase-PCR and cDNA amplification by PCR with primers *orfHA* (F1 and R1) **(A)**, *shf* (613 pb) **(B)**, and *aaiC* (215 pb) **(C)**. MM: 1kb Plus DNA Ladder. Agarose gel (1%) stained with ethidium bromide.

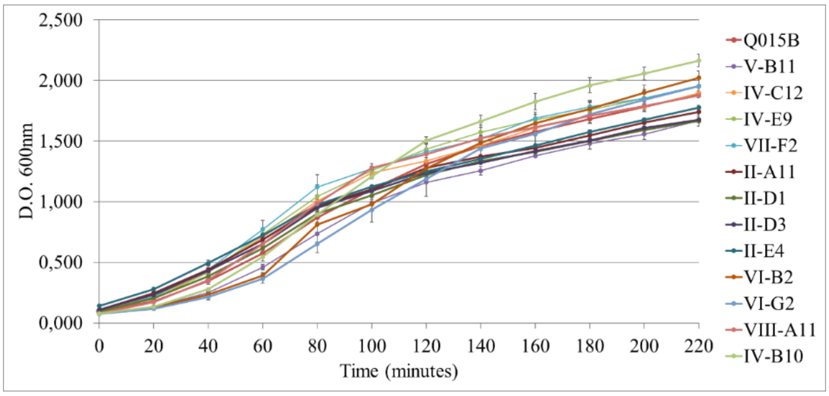

FIGURE S3. Growth curves. Cultures grown overnight in LB medium containing kanamycin were adjusted to the same density based on OD_{600} diluted 1:100 and cultivated in LB medium containing kanamycin at 37°C with agitation at 150 r.p.m. The OD_{600} was measured every 0.5 h until 220 minutes have been completed. This experiment was performed in triplicate.

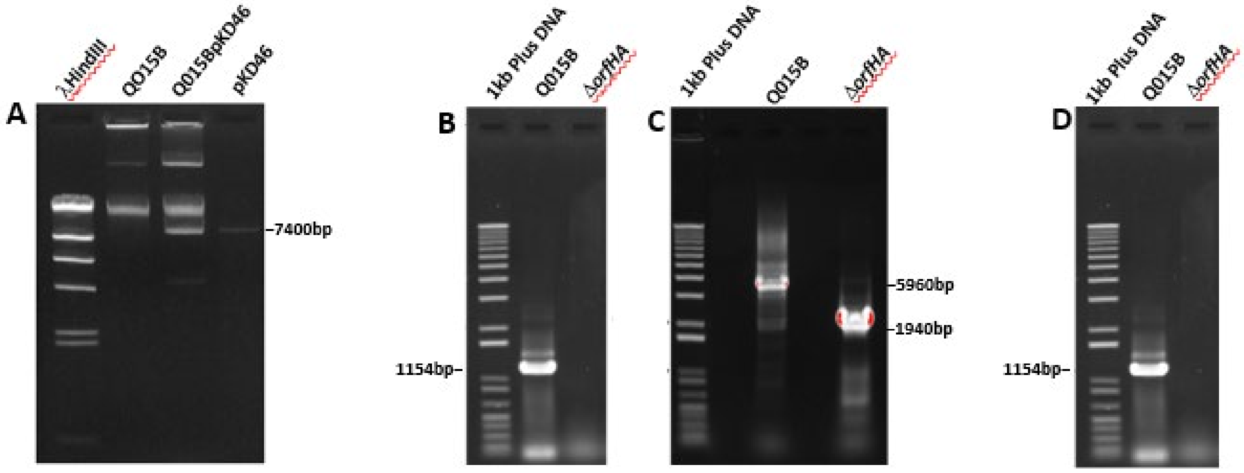

FIGURE S4. Construction of the orfHA mutant. (A) Plasmids from EAEC Q015B and pKD46Cm (7400 bp); (B) and (C) *orfHA* deletion was confirmed by PCR using the ORF's internal primers (F1 and R1), yielding a 1154 fragment in the wild-type strain and no PCR product in the Δ*orfHA* mutant, and ORF's external primers (F3 and R3) resulting in a 5960 bp fragment in the wild-type strain and a 1940 bp fragment in the Δ*orfHA* mutant; (D) RT-PCR of *orfHA* transcripts with Q015B and Q015BΔ*orfHA* mutant strains. Agarose gel (1%) stained with ethidium bromide.

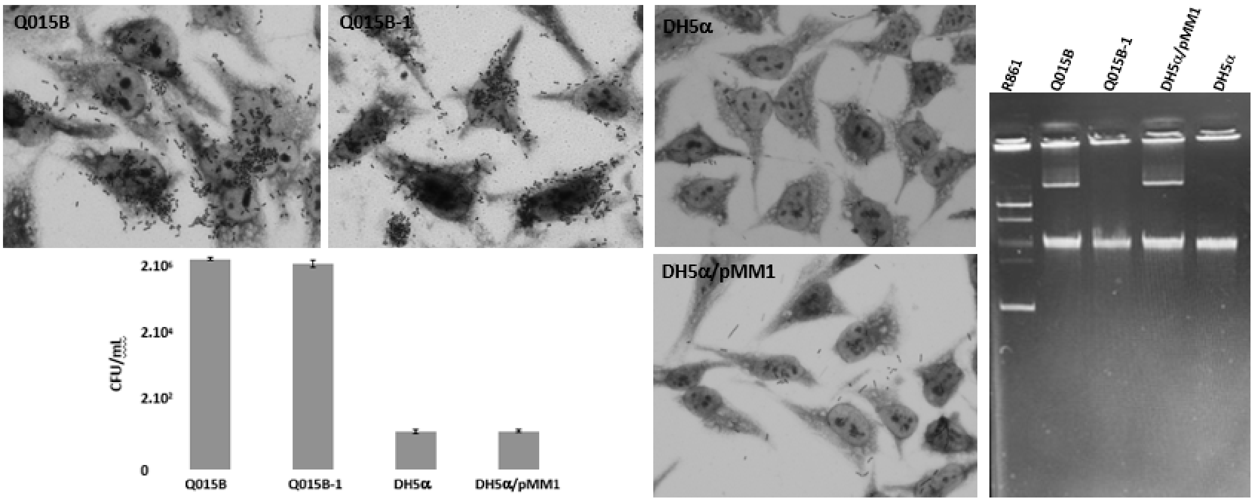

FIGURE S5. Adhesion of Q015B, Q015B-1, *E. coli* DH5α and transformant *E. coli* DH5α/pMM1 strains to HeLa cells. Magnification, x1,000. Quantitative adherence of Q015B, Q015B-1, *E. coli* DH5α and DH5α/pMM1 strains. Plasmid profiles of Q015B, Q015B-1, DH5α/pMM1, and *E. coli* DH5α strains. Bacterial strains were incubated with HeLa cell monolayers for 3 h on glass coverslips in a 24-well microtiter plate in DMEM. Plasmids were isolated from strains by alkaline lysis, separated on a 0.8% agarose gel. R861 strain carrying plasmids of known molecular sizes (Threlfall et al., 1986).

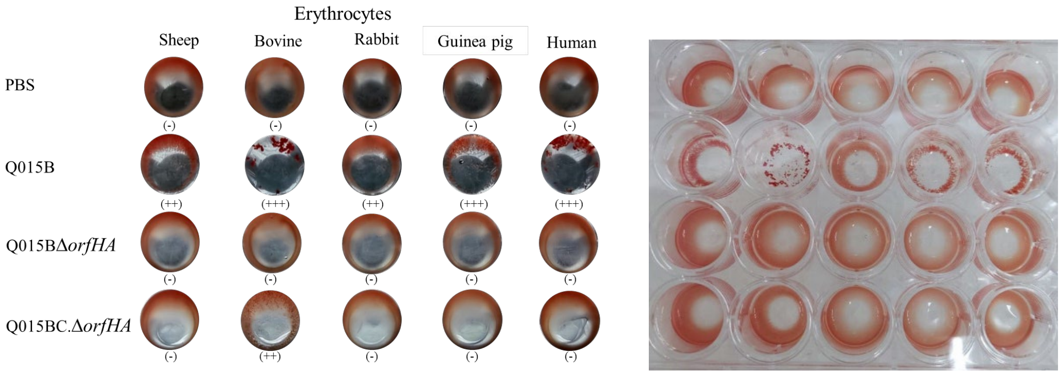

FIGURE S6. HA phenotypes of EAEC Q015B and derivative strains. Mannose-resistant HA with erythrocytes from different animal species. Line 1: Negative control (PBS). Line 2: Q015B (positive). Line 3: Q015BΔ*orfHA* Line 4: Q015BC.Δ*orfHA*. Column 1: Sheep erytrocytes. Column 2: Bovine erytrocytes. Column 3: Rabbit erytrocytes. Column 4: Guinea pig erytrocytes. Column 5: Human erytrocytes. HA was labeled: (+) aggregates of erythrocytes visible only under light microscope; (++), aggregates visible with the unaided eye; +++, large aggregates; (−) no aggregates (negative).
